# Distributed cerebellar plasticity implements generalized multiple-scale memory components in real-robot sensorimotor tasks

**DOI:** 10.3389/fncom.2015.00024

**Published:** 2015-02-25

**Authors:** Claudia Casellato, Alberto Antonietti, Jesus A. Garrido, Giancarlo Ferrigno, Egidio D'Angelo, Alessandra Pedrocchi

**Affiliations:** ^1^NeuroEngineering And Medical Robotics Laboratory, Department Electronics, Information and Bioengineering, Politecnico di MilanoMilano, Italy; ^2^Brain Connectivity Center, IRCCS Istituto Neurologico Nazionale C. MondinoPavia, Italy; ^3^Department of Computer Architecture and Technology, University of GranadaGranada, Spain; ^4^Department Brain and Behavioral Sciences, University of PaviaPavia, Italy

**Keywords:** cerebellar model, neurorobot, motor learning, distributed plasticity, long term plasticity

## Abstract

The cerebellum plays a crucial role in motor learning and it acts as a predictive controller. Modeling it and embedding it into sensorimotor tasks allows us to create functional links between plasticity mechanisms, neural circuits and behavioral learning. Moreover, if applied to real-time control of a neurorobot, the cerebellar model has to deal with a real noisy and changing environment, thus showing its robustness and effectiveness in learning. A biologically inspired cerebellar model with distributed plasticity, both at cortical and nuclear sites, has been used. Two cerebellum-mediated paradigms have been designed: an associative Pavlovian task and a vestibulo-ocular reflex, with multiple sessions of acquisition and extinction and with different stimuli and perturbation patterns. The cerebellar controller succeeded to generate conditioned responses and finely tuned eye movement compensation, thus reproducing human-like behaviors. Through a productive plasticity transfer from cortical to nuclear sites, the distributed cerebellar controller showed in both tasks the capability to optimize learning on multiple time-scales, to store motor memory and to effectively adapt to dynamic ranges of stimuli.

## Introduction

In order to develop a comprehensive theory of learning, it is crucial to define a causality chain linking neural signals, plasticity mechanisms, neural circuits and behavioral learning (Cheron et al., 2013). Cerebellar-mediated learning ranges from associative conditioning of discrete behavioral responses to on-line adaptation in voluntary and reflex movement control (Ito, 1982), driving acquisition, tuning, extinction and consolidation of motor skills.

In order to learn and store information in internal models of movement so to act as a predictive controller, the cerebellum is thought to employ long-term synaptic plasticity: Long-Term Depression (LTD) and Long-Term Potentiation (LTP). The plasticity at the Parallel Fibers-Purkinje Cells (PF-PC) synapses has classically been assumed to subserve this function (Marr, 1969). However, multiple processes, with different learning rates, may contribute to these mechanisms (Smith et al., 2006; Lee and Schweighofer, 2009; Shadmehr et al., 2010) and PF-PC single plasticity cannot account for the broad dynamic ranges and multiple time scales of cerebellar adaptation. One hypothesis is that the cerebellum learns basically on two time scales ascribable to two anatomical sites: the cerebellar cortex operates as a fast learning module while deeper structures operate as a slow learning module where the motor skill is transferred and consolidated into more persistent memory (Medina et al., 2001). Indeed, the activity of the Deep Cerebellar Nuclei (DCN) can be modulated and DCN spike times are strongly correlated with memory acquisition (Zhang and Linden, 2006). However, there have been few physiological studies on long-term plasticity in DCN and on their roles in motor learning paradigms. Cerebellar cortical and nuclear plasticities have been proposed to be involved and complementary in controlling cerebellar learning in EyeBlink Classical Conditioning (EBCC) (Bracha et al., 1998; Medina and Mauk, 2000; Medina et al., 2000) and in Vestibulo-Ocular Reflex (VOR) (Burdess, 1996; Ito, 1998; Masuda and Amari, 2008). Indeed, inactivation of cerebellar cortex (Attwell et al., 2001), cerebellar nuclei (Attwell et al., 2002) or Inferior Olive (IO) (Welsh and Harvey, 1998) all prevent acquisition skills. There are several possible molecular and cellular mechanisms that could underlie adaptation of the vestibulo-ocular reflex and eyeblink conditioning. Behavioral observations showed common and robust mechanisms between EBCC and VOR tasks: slow and fast complementary adaptation processes, spontaneous recovery of the original response and faster relearning due to consolidation mechanisms. However, causal relationships between particular cellular processes and individual components of a learned behavior have not been demonstrated unequivocally (De Zeeuw and Yeo, 2005).

One complementary approach to the experimental and behavioral one to better understand the mechanisms of the cerebellum information processing is to make computational models of the cerebellum network and to test them in paradigms as close as possible to the neurophysiological ones. Different simplified cerebellar models based on the Adaptive Filter Model derived from the Marr-Albus Motor Learning Theory have been developed (Marr, 1969; Albus, 1971; Tyrrell and Willshaw, 1992; Ito, 1997; Lepora et al., 2010), in few studies also translated into spiking neural networks (Yamazaki and Tanaka, 2007; Yamazaki and Nagao, 2012), and tested in computational simulations of EBCC, VOR and upper-limb tracking tasks. In these models, learning occurred as long-term PF-PC single plasticity.

Very recently (Garrido et al., 2013), beside the PF-PC cortical plasticity, the cerebellum model was endowed with biologically plausible plastic mechanisms at two additional synaptic sites of DCN: Mossy Fibers-DCN (MF-DCN) and PC-DCN (Hansel et al., 2001; Gao et al., 2012). This 3-site cerebellar model, as a general computational scheme, was tested in a tracking task only in simulations. *In-silico* simulations are always a first important test bench of new features of controllers but they can only partially be used to show the real behavior of a computational model. The literature has already discussed this point and interesting works have been proposed to check the use of neural-inspired control models acting into real-world conditions by being embedded into a controller of a robot (Voegtlin and Verschure, 1999; Hofstotter et al., 2002; McKinstry et al., 2006; Lenz et al., 2009; Trhan, 2010; Batllori et al., 2011; Yamazaki and Igarashi, 2013). Indeed, modeling the cerebellar structure and embedding it into the control of a real robot immersed into real-world conditions is a key approach to associate the detailed model of neuronal connectivity and synaptic plasticity with behavioral functionalities. Experiments with real robots allow the exploration of the robustness and generalization capability of the controlling model (Verschure and Voegtlin, 1998). Specific experimental paradigms have been already proposed in the literature to highlight specific features of the computational model used in the robotic control, for example Yamazaki and coworkers (Yamazaki and Igarashi, 2013) designed a ball intercept robotic task in order to test the timing properties of a single-plasticity spiking cerebellar controller.

In this context, we have tested into realistic sensorimotor tasks the learning skills of the 3-site distributed plasticity cerebellar model, embodied in a robot acting and sensing in real-time in real environment (*neurorobot*). In order to focus our tests on the learning properties of the cerebellar network model, the robot has been acting in two experimental protocols of different nature, selected to mimic typical cerebellum-mediated neurophysiological paradigms: an associative Pavlovian task, as the EBCC, and a VOR. Very often the models using cerebellar principles, more or less detailed and realistic, have been designed specifically for one single task (Van der Smagt, 2000; Day et al., 2006; Yamamoto et al., 2007; Thompson and Steinmetz, 2009; Clopath et al., 2013), while the real cerebellum is good at learning a wide variety of tasks, going from stimuli associations to adaptive sensorimotor transformations and coordination.

The Pavlovian associative task is learned along with repeated presentation of paired stimuli, a Conditioned Stimulus (CS, like a tone) followed by an Unconditioned Stimulus (US, like an air-puff, eliciting the eye-blink reflex). The cerebellum learns to produce a Conditioned Response (CR, like an eye-blink, anticipating the US onset) (Medina et al., 2000). The VOR produces eye movements that stabilize images on the retina compensating head movements. The EBCC requires a fine timing control, whereas the VOR requires a continuous timing and gain control (Yamazaki and Nagao, 2012).

We have compared the performances of the 3-site distributed plasticity model with the basic one that implements only the PF-PC learning rule. Each task, repeated varying the provided stimuli or the perturbation patterns, was tested and then re-tested after extinction. The main goal is to seek the real-world behavioral outcomes generated by the neural mechanisms modeled into the cerebellar controller, in order to deepen the roles of the plasticity sites and their interaction along multiple learning stages.

## Materials and methods

### Neurorobot

The robot was a Phantom Premium 1.0 (SensAble™), with 3 degrees of freedom, equipped with digital encoders and controllable by torque commands. It was integrated with an optical tracking system, a VICRA-Polaris (NDI™), acquiring marker-tools at 20 Hz. The controller, ad-hoc developed in C++, exploited the low-level access provided by the Haptic Device Application Programming Interface, sending the torque signals to the joints by servo loops (HDCALLBACKS) executed in high-priority threads at 1 kHz. For the tracking device, the low-level libraries from Image-Guided Surgery Toolkit (http://www.igstk.org/), based on Request-Observer-patterns, were used to acquire the visual information. The cerebellar adaptive module was embedded into the C++ controller.

### Protocols

**Figure 1:**
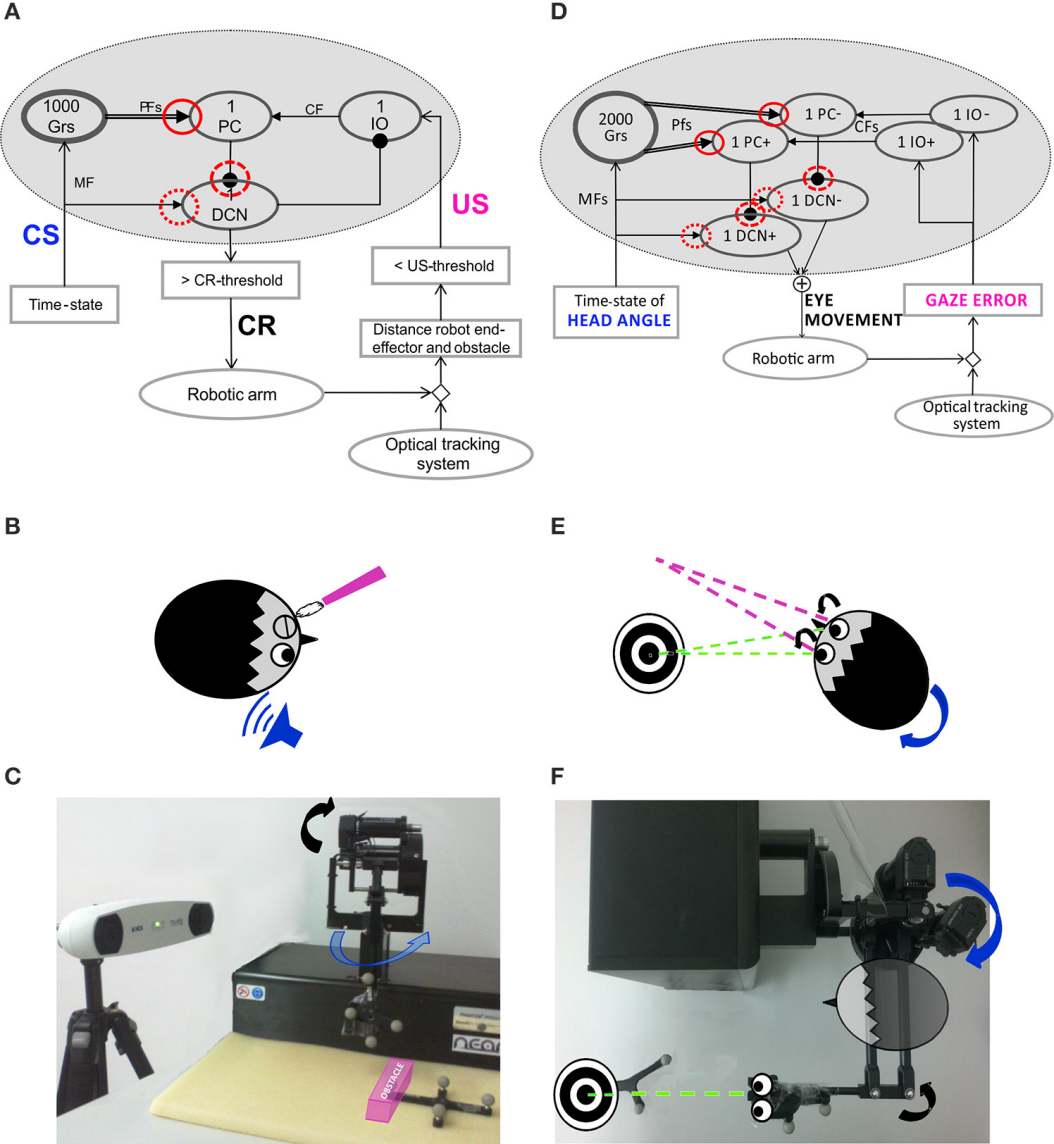
**Embodied cerebellar model and set-up**. **(A)** Cerebellar model embedded into the neurorobot, with EBCC-specific input and output signals. The red circles represent the plasticity sites: straight line the PF-PC synapses; dot line and dashed line the MF-DCN and PC-DCN synapses, respectively, activated only within the 3-plasticity model. Arrows represent excitatory connections, whereas dot-arrows inhibitory connections. The EBCC-like Pavlovian task is reproduced into the robotic platform by defining the onset of the US stimulus based on the distance between the moving robot end-effector and the fixed obstacle placed along the trajectory (US-threshold), detected by the optical tracker. CS, fed into the CF pathway, represents the system time-state, decoded by the GR layer. CS and US coterminate (“delay EBCC”). The DCN triggers the conditioned response (CR). **(B)** Human-like EBCC task. **(C)** Robotic set-up reproducing the Pavlovian EBCC-like task. **(D)** Cerebellar model with VOR-specific input and output signals. The red circles represent the plasticity sites: straight line the PF-PC synapses; dot line and dashed line the MF-DCN and PC-DCN synapses, respectively, activated only within the 3-plasticity model. Arrows represent excitatory connections, whereas dot-arrows inhibitory connections. The VOR is reproduced into the robotic platform by using the second joint of the robotic arm as the head (imposed rotation) and the third joint (determining the orientation of the second link, on which the green laser is placed) as the eye. The disalignment between the gaze direction (i.e., second link orientation) and the environmental target to be looked at is computed through geometric equations from the optical tracker recording. Head vestibular stimulus represents the system time-state, decoded by the GR layer. The gaze error is fed into the CF pathway, the DCN modulate the compensatory eye movement. **(E)** Human-like VOR task. **(F)** Robotic set-up reproducing the VOR task.

The **Pavlovian EBCC-like protocol** was reproduced as a collision-avoidance task in real-robot (Figures 1A–C). The robotic arm was moving on a pre-defined straight trajectory (followed thanks to joint torques computed through a Proportional-Derivative feedback controller, given the desired joint kinematics and the actual joint kinematics). A fixed obstacle was placed along that path. The US was a step lasting 200 ms; the rise front was triggered through the tracking system, when the distance between obstacle-vertex and robot end-effector underwent a pre-defined threshold (US-threshold, collision risk). Thus, this threshold determined the Inter-Stimuli-Interval (ISI). US-signal reached PCs through Climbing Fibers (CFs). The CS on the MFs (passage of time from trial onset) was decoded into the GRanular (GR) layer. In order to reproduce the “delay EBCC,” i.e., the two stimuli on MF and CF pathways co-terminated in each trial, the input from MFs was made silent from the end of US till the end of the trial. Each trial lasted 1 s.

The DCN response, anticipated with respect to the US-onset, modulated the upcoming US-based signal, ranging between 0 and 1, through an inhibitory connection from DCN to IO, i.e., in each trial, *IO* = 1 − *DCN*(*t_USonset_*). Stronger was the anticipated eyelid protecting the eyes from US, less powerful was the US-related signal arriving on the PC from IO (Medina et al., 2000).

The CR generation, by thresholding the DCN activity [*DCN*(*t*) ≥ 0.9], triggered a pre-programmed 15° triangular increase of the desired angle for the second joint of the moving robotic arm, so the obstacle was vertically overstepped.

The test was made up of two sessions (session1 and session2). Each session consisted of 80 trials of acquisition (CS-US paired presentation) directly followed by 20 trials of extinction (CS-alone, with CS lasting 600 ms).

In order to validate the robustness of the embedded cerebellar controller, different stimuli patterns, i.e., three US-thresholds, were defined so as to generate three average ISIs within a physiologically effective range (Shibuki et al., 1996): US-th_1_ = 80 mm; US-th_2_ = 105 mm; US-th_3_ = 120 mm. For each US-threshold, 20 tests were carried out. DCN activity was analyzed; as the timing nature of the protocol, we focused on the maximum DCN activity achieved within each trial.

The **VOR** was reproduced by using the 2nd joint as the head, on which a desired joint displacement was imposed, and the 3rd joint as the eye motion driven only by the cerebellar module. The set-up was arranged so that the two involved joints (2nd and 3rd) moved on a horizontal plane (Figures 1D–F). The visual error, thanks to the tracking system, was computed as the disalignment angle between the actual gaze, i.e., the orientation of the second link of the robot, and the desired one aligned with the fixed object to be looked at (identified by a markers-tool). The normalized value of this visual error was sent to the IO corresponding to the actual error sign (IO+/IO−). Two PCs (one receiving IO+ and one receiving IO−) inhibited DCN+ and DCN− activity, respectively. The net activity of DCNs was proportionally translated, through a fixed gain set to 0.065 Nm, into a net torque on the 3rd joint at each time sample (Luque et al., 2011). The neural controller architecture and computational principles were designed as for the Pavlovian task, except that at the input stage two IO subgroups coding the error directions (sign), and, accordingly, at the output stage, two DCN subgroups coding the motor command directions (sign) were included. It is biologically plausible (Georgopoulos et al., 1986) and consistent with the different nature of the two cerebellar paradigms: the stimuli-based discrete EBCC and the continuous direction-dependent VOR. Unlikely the EBCC-like task, no inhibitory connection between DCNs and IOs was modeled, because the DCN activity itself, by changing the outcome gaze angle, directly affected the gaze error signal coded by the IOs.

The test was made up of two VOR sessions (session1 and session2) with fixed target. Each VOR session consisted of 40 trials of acquisition by imposing a pre-defined head rotation, directly followed by 20 extinction trials (head turn null).

In order to validate the robustness of the embedded cerebellar controller, different vestibular stimulus patterns, i.e., three Head Rotation (HR) profiles, were set: HR_1_ = 25° in 2 s, HR_2_ = 30° in 2 s, HR_3_ = 35° in 2 s. For each HR, 15 tests were carried out.

In order to check the capability to rapidly face changes of the stimulus, for each cerebellar controller, a second test was carried out. It reproduced initially the same condition as in the VOR session1 with HR_1_ = 25° in 2 s, but during the steady plateau of the network outcome (at the 35th trial of acquisition), a gain-up stimulus was provided: the head rotation was increased 1.5 times, from 25° to 37.5°, and imposed for other 15 trials. Thus, the test was made up of 50 repetitions.

Gaze error and DCN activity were analyzed; since the protocol required a continuous shape modulation of the motor response, we focused on the Root Mean Square (RMS) of the net DCN activity (taking into account the net activity, DCN+ and DCN−) within each trial.

### Cerebellum model

In this work, we adapted the cerebellar model developed in (Garrido et al., 2013). The model represents a theoretical abstraction of the laying physiological mechanisms, it is inspired by the cerebellum neurophysiological mechanisms but the univocal correspondences are not straightforward.

Neuronal signals traveling represent firing rates for the associated neuron or population of neurons, so that all information is assumed rate-coded. Shortly, MF activity, the “context” information, is represented by a constant activity and the GR layer circuit is capable of generating not-recurrent time-evolving states, in each trial, thus univocally identifying the passage of time (Yamazaki and Tanaka, 2007). This procedure formally corresponds to a labeled-line coding scheme. One different state for each time sample is generated through a sequential activation of PFs within each trial. Hence, the number of PFs (axons of GRs) depends on the movement duration.

The DCNs integrate the excitatory activity coming from MFs and the inhibitory activity coming from PCs (1).

(1) $$DCN_{i}\left(t\right)\;=\;W_{MF\text{-}DCN_{i}}\left(t\right)-Pur_{i}\left(t\right)\;\cdot \;W_{PC_{i}\text{-}DCN_{i}}\left(t\right)\;i=1,\;2$$

Where *DCN*_*i*_(*t*) represents the activity of the DCN associated with the agonist (*i* = 1, i.e., +) or antagonist (*i* = 2, i.e., −) actuators, *W*_*MF*-*DCN*_*i*__(*t*) is the synaptic strength of the MF-DCN connections at the *i*-th actuator, and *W*_*PC*_*i*_-*DCN*_*i*__ (*t*) is the synaptic strength of the PC-DCN connections at the *i*-th actuator. For the Pavlovian task, only one actuator is contemplated (*i* = 1). *Pur*_*i*_(*t*) is the current activity coming from the associated PC (2).

(2) $$Pur_{i}\left(t\right)=f_{i}(PF(t))\;i=1,\;2$$

Where *Pur_i_*(*t*) represents the firing rate of the PCs associated with the *i*-th actuator and *f_i_* associates each granular layer state (i.e., one active PF) with a particular output firing rate at the *i*-th PC.

The Purkinje cells receive error from CFs, coming from IOs, and state information from GRs, and work as a state-error correlator (3).

(3) $$\begin{array}{l} \Delta W_{PF_{j}\text{-}PC_{i}}\left(t-\tau \right)\\ =\left\{ \begin{array}{l} \frac{{}^{1}LTP_{max}}{{\left(\varepsilon_{i}(t)+1\right)}^{\alpha }}{-}^{1}LTD_{max}\;\cdot \;\varepsilon_{i}\left(t\right)\;if\;PF_{j}\;is\;active\;at\;t\\ \;0\;otherwise \end{array}\right. \end{array}$$

Where Δ*W*_*PF*_*j*_-*PC*_*i*__(*t*) is the weight change between the *j*-th PF and the *i*-th PC associated with the agonist actuator (*i* = 1) or with the antagonist actuator (*i* = 2); *j* is the number of PFs, equal to the time samples within each task repetition; ε_*i*_(*t*) is the current activity coming from the associated *i*-th CF, expressed from 0 to 1. ^1^*LTP*_*max*_ and ^1^*LTD*_*max*_ are the maximum LTP and LTD values and α is the LTP decaying factor (set at 1000 in order to allow a fast decrease of LTP and prevent early plasticity saturation). With respect to the model in (Garrido et al., 2013), we introduced the τ term, so modeling the physiological delay of the neural circuit, which ranges from 50 to 150 ms (Gerwig et al., 2005); it means that the weight update takes into account the PF activity preceding the error-related signal carried by CFs. We set τ equal to 100 ms.

The weights at the DCN plastic sites evolve as the learning rules in (4) and (5).

(4) $$\begin{array}{l} \Delta W_{MF\text{-}DCN_{i}}(t)=\frac{{}^{2}LTP_{max}}{{\left(Pur_{i}\left(t\right)+1\right)}^{\propto }}{-}^{2}LTD_{max}\\ \;\cdot \;Pur_{i\;}\left(t\right)\;i=1,\;2 \end{array}$$

Where Δ*W*_*MF*-*DCN*_*i*__(*t*) represents the weight change between the MF and the target DCN associated with the *i*-th actuator, ^2^*LTP*_*max*_ and ^2^*LTD*_*max*_ are the maximum LTP and LTD values and α is the LTP decaying factor (= 1000).

(5) $$\begin{array}{l} \Delta W_{PC_{i}\text{-}DCN_{i}}\left(t\right)={\;}^{3}LTP_{max}\;\cdot \;Pur_{i}{\left(t\right)}^{\alpha }\\ \;\cdot \left(1-\frac{1}{{\left(DCN_{i}\left(t\right)+1\right)}^{\propto }}\right){-}^{3}LTD_{max}\\ \;\cdot \left(1-Pur_{i}\left(t\right)\right)\;i=1,\;2 \end{array}$$

Where Δ*W*_*PC*_*i*_-*DCN*_*i*__(*t*) is the synaptic weight adjustment at the PC-DCN connection reaching the DCN associated with the *i*-th actuator, *Pur*_*i*_(*t*) is the current activity coming from the associated PC, *DCN*_*i*_(*t*) is the current DCN activity, ^3^*LTP*_*max*_ and ^3^*LTD*_*max*_ are the maximum LTP and LTD values and α is the LTP decaying factor (= 1000). All the weights were initialized to 1.

Previous findings suggest a concrete but speculative mechanism for inducing LTP and LTD at MF-DCN synapses that is consistent with Purkinje cell control of plasticity at these sites (Llinás and Mühlethaler, 1988; Medina and Mauk, 1999; Ozgur et al., 2006; Pugh and Raman, 2006; Zhang and Linden, 2006). In particular, MF-DCN synapses may increase in strength when coactive during the high levels of calcium likely to exist during transient decreases in Purkinje cell activity and decrease in strength when active during lower levels of calcium, as may occur during strong inhibitory input from Purkinje cells.

The cerebellar controller was embedded into the whole control system, with input and output customized on the task (Figures 1A,D).

The *LTP_*max*_* and *LTD_max_* of each plasticity site could be tuned. In the 1st plasticity site (PF-PC), ^*1*^*LTP_max_* has to be lower than ^*1*^*LTD_max_*, otherwise LTP, constantly generated when a state-related activity comes from GRs, could counterbalance and nullify LTD effects. Moreover, to maintain the stability of the learning process, *LTP_max_* and *LTD_max_* values of the other two plasticity rules (^2^*LTP_max_*,^2^*LTD_max_*, ^3^*LTP_max_*, and ^3^*LTD_max_*) have to be lower than those defined at the PF-PC synapses.

We have explored different LTP and LTD values, evaluating their effect on the acquisition effectiveness and rate, on the late acquisition stability and on the extinction effectiveness and rate. We have tested a delay-EBCC task in computational simulations, with an EBCC session made up of 80 trials of acquisition (CS-US pairs) and 20 trials of extinction (CS-alone). The CS was provided as a constant activity, activating one different PF each 1 ms, US as a step signal whose amplitude was reduced from the maximum (= 1) depending on the ongoing DCN activity at the US onset instant (as described above in the *Protocols* section). US lasted 200 ms and occurred after 200 ms from each trial onset (each trial lasted 400 ms).

**Figure 2:**
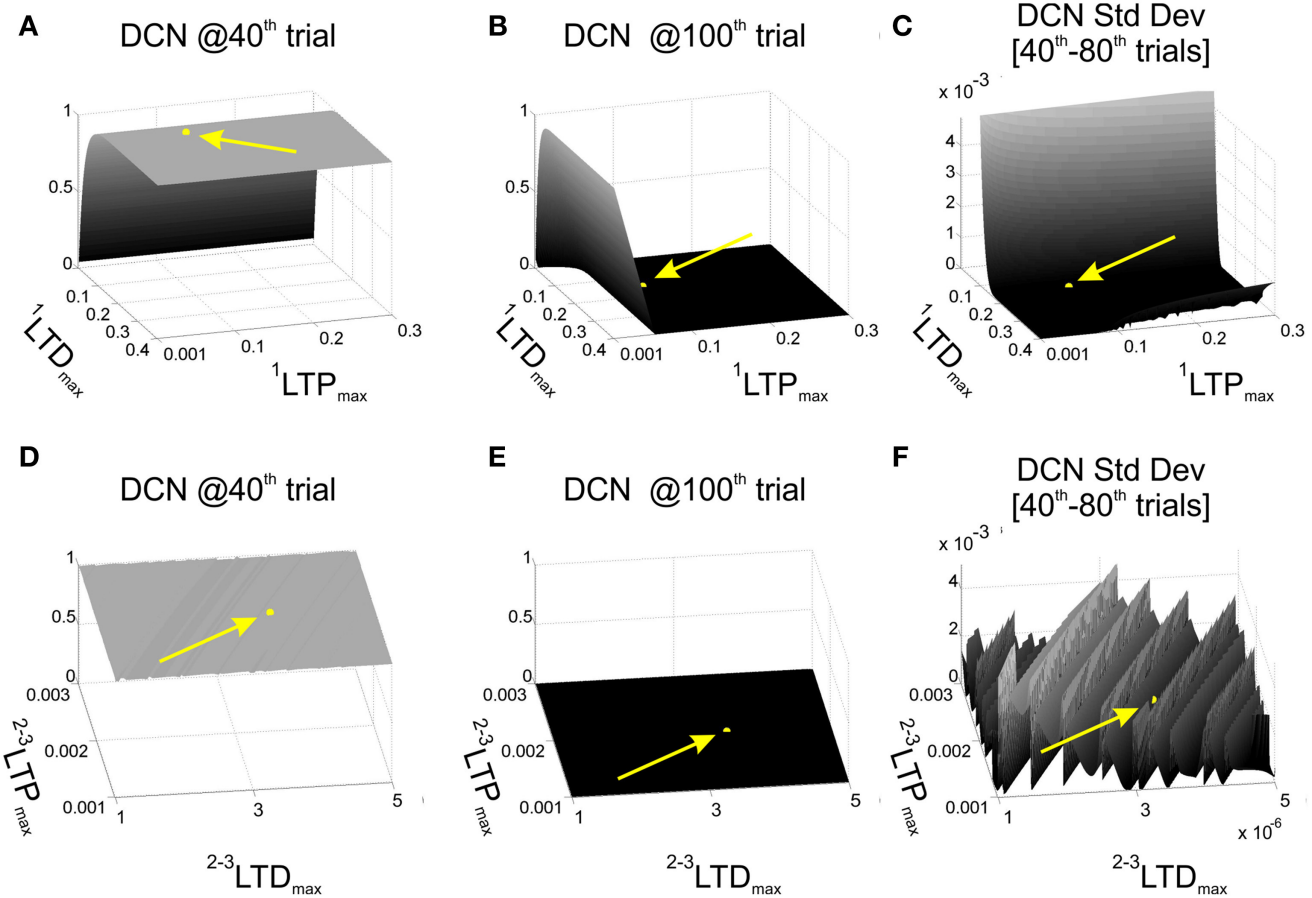
**Cerebellar model parameters: LTD and LTP**. EBCC task (80 trials of acquisition + 20 trials of extinction) in computational simulations in which *LTD_max_* and *LTP_max_* of the plasticity rules were varied, testing 1200 parameters combinations. First row reports the network performance when the task was controlled by the 1-plasticity model and varying ^1^*LTD_max_* and ^1^*LTP_max_*. **(A)** Maximum DCN output at the 40th trial (achieved acquisition); **(B)** maximum DCN output at the 100^th^ trial (late extinction); **(C)** maximum DCN output standard deviation from 40th to 80th trials (late acquisition stability). Once fixed ^1^*LTD_max_* and ^1^*LTP_max_*, the same performance parameters **(D–F)** were evaluated when the task was controlled by the 3-plasticity model, varying the *LTD_max_* and *LTP_max_* of the other two plasticity sites (^2^*LTD_max_*, ^2^*LTD_max_*, ^3^*LTD_max_*, ^3^*LTP_max_*). The yellow arrows and points highlight the position of the selected LTP and LTD parameters.

For each combination (1200) of *^*1*^LTP_max_* and *^*1*^LTD_max_*, we performed one computational simulation in which the behavior was controlled by the single-site model (*W_*MF*-*DCN*_* and *W_PC-DCN_* fixed at 1). We established some significant instants during the EBCC session, to evaluate the effects of the *^*1*^*LTP*_max_* and *^*1*^*LTD*_max_* values on the learning process. In details, DCN activity at the 40th trial was used as index of acquisition, since after 40 repetitions the cerebellum should have learned the stimuli association and achieved a steady maximum output activity. As index of acquisition stability, the DCN activity standard deviation from 40th to 80th trials was computed, and it was supposed to be minimized. Finally, the DCN activity at the 100th trial provided us with an index about the skill of “reverse learning” (extinction), which was supposed to be a fast and effective process. Thus, we set *^*1*^*LTP*_max_* and *^*1*^*LTD*_max_* as a compromise between maximum acquisition level, minimum oscillations during the plateau in late acquisition, and maximum extinction effectiveness. Among the acceptable combinations, we hence set *^*1*^*LTP*_max_* = 0.1 and *^*1*^*LTD*_max_* = 0.15 (Figures 2A–C).

Further, we activated the other two plasticity sites into the controller and we repeated the tests, exploring different *LTP_max_* and *LTD_max_* values at MF-DCN and PC-DCN, imposing an analogous dynamics to the two learning rules (^2^*LTP_max_* = ^3^LTP_*max*_ and ^2^*LTD*_*max*_ = ^3^LTD_*max*_) (Garrido et al., 2013). We computed the same 3 indexes on the DCN activity (value at the 40th trial, standard deviation 40th–80th trials, and value at the 100th trial). The goal was to maintain the same properties achieved in the tests driven only by the PF-PC plasticity. Indeed, the slower deep nuclear synapses should not significantly affect the overall behavior during a single session started from a naïve state, but rather during a re-testing phase. Among the acceptable combinations, we hence set ^2^*LTP_max_* = ^3^*LTP_max_* = 2·10^−3^ and ^2^*LTD_max_* = ^3^*LTD_max_* = 3.5·10^−6^ (Figures 2D–F). Indeed, the multi-rate learning modeling suggests that the role of the slower nuclear dynamics should emerge in longer timeframes calling for consolidation mechanisms, as the ones designed for the robot described above (multi-session EBCC and VOR).

Therefore, afterwards the tuning of the model parameters by simulations from which *LTP_max_* and *LTD_max_* parameters of all the three sites were defined, we have investigated whether, in real-robot multiple-session protocols, the 3-site distributed model determined a different learning behavior and how these potential differences were related to any interactions of the synaptic weight changes at cortical and nuclear sites.

### Data analyses

All tests were performed embedding the 1-plasticity cerebellum model (PF-PC) and the 3-plasticity model (PF-PC, MF-DCN, PC-DCN) into the robotic controllers.

For the Pavlovian task, the model performances were evaluated by computing the average gradient of the maximum DCN activity during acquisition trials (1st–40th trials) in session1 (ΔDCN_s1_) and in session2 (ΔDCN_s2_) and by calculating the mean anticipation value of the DCN activity onset, generating CR, with respect to the US onset (latency) along all the two-session trials.

For the VOR task, the model performances were evaluated computing the average gradient of the RMS DCN activity during acquisition trials (1st–10th trials) in session1 (ΔDCN_s1_) and in session2 (ΔDCN_s2_).

The One-Way ANOVA test was used to check whether each model showed a comparable performance across the different experimental conditions of each task (3 levels of ISI and 3 levels of HR).

For each parameter quantifying the learning performance, the *t*-test for two independent populations was applied to highlight any differences between 1-plasticity and 3-plasticity behaviors, for each experimental condition separately (ISI_1_, ISI_2_, and ISI_3_; HR_1_, HR_2_, and HR_3_).

In all tests, the level of statistical significance was preset to *p* < 0.01. Unless otherwise stated, all results are indicated as mean ± standard deviation.

## Results

### Pavlovian task

**Figure 3:**
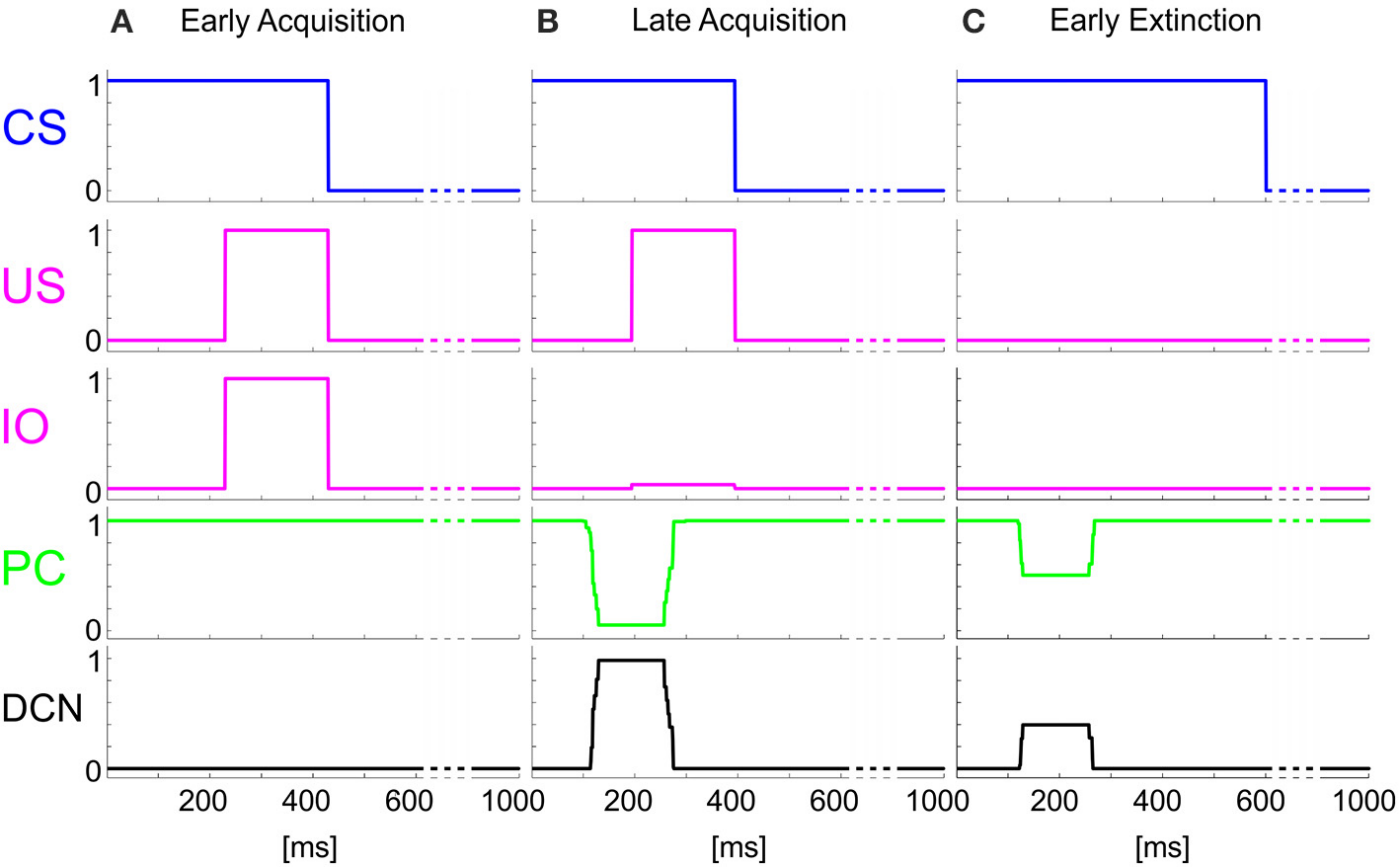
**Real-robot Pavlovian task**. Three exemplificative trials of the session1 (80 trials of acquisition + 20 trials of extinction) of Pavlovian task carried out by the neurorobot are described by displaying the provided CS and US, IO, PC and DCN activity. **(A)** 1st trial, **(B)** 80th trial, **(C)** 85th trial.

The time-evolving states were decoded into the granular layer. From granule cells, activity was transmitted to the PC and in parallel excited the DCN. The US-related pattern reached the Purkinje cell when US-threshold was detected. The Purkinje cell in turn inhibited the DCN. At the beginning of the acquisition phase, the Purkinje cell was spontaneously active, supplying tonic inhibition to the DCN (Figure 3A). After acquisition, PC activity was decreased; summing up all the presynaptic (constant or plastic) inputs to DCN, DCN neurons began to fire strongly before the onset of the US as neurorobot acquired the CR (Figure 3B). Then during extinction trials, PC activity was progressively re-increased; and DCN did not produce CR anymore (Figure 3C).

In each condition (three different US thresholds), the ISI came out not perfectly constant, both across trials of the same test and across the 20 tests (ISI_1_ 1-plast: 349 ± 16; ISI_1_ 3-plast: 350 ± 16; ISI_2_ 1-plast: 478 ± 21; ISI_2_ 3-plast: 493 ± 34; ISI_3_ 1-plast: 524 ± 17; ISI_3_ 3-plast: 516 ± 17 ms). This variability was related to the noise due to the inertial components of the robot and to the tracking system refresh. In each condition, no statistical differences came out between the resulting ISI which 1-plasticity and 3-plasticity models were subjected to. Thus, the necessary prerequisite about the same experimental conditions between the two cerebellar models was verified, so allowing us to ascribe possible differences between their behavioral outcomes to the different neural mechanism functioning.

The One-Way ANOVA tests confirmed the robustness and consistency of each cerebellar model behavior in the three experimental conditions. Both for the 1-plasticity model and for the 3-plasticity model, ISI value did not affect the learning rates, neither the CR latency (overall mean CR latency 1-plast: 66 ± 19 ms; overall mean CR latency 3-plast: 63 ± 23 ms). Only for the 3-plasticity model in session1, the ISI factor resulted significant on the ΔDCN_s1_ (*F* = 11.9; *p* = 0.00005).

**Figure 4:**
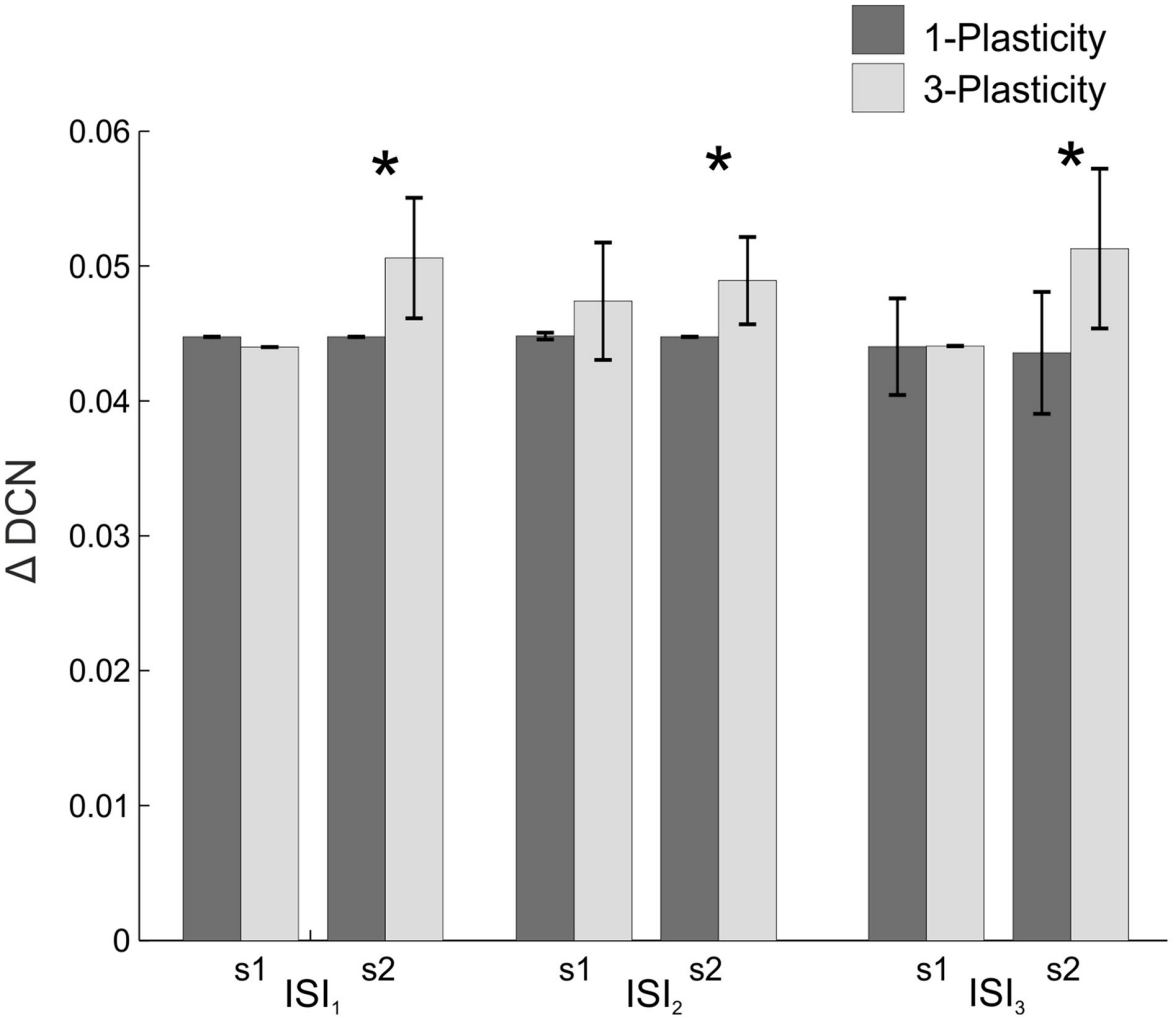
**Real-robot two-session Pavlovian task: models comparison**. The histogram reports the performances (ΔDCN) of the 1-plasticity and 3-plasticity models in the three EBCC conditions (ISI_1_, ISI_2_, and ISI_3_) in both sessions (s1 and s2). Bars indicate the mean across the 20 tests and the relative standard deviation. ^*^Corresponds to significant statistical difference between the two cerebellar models (*p* < 0.01).

**Figure 5:**
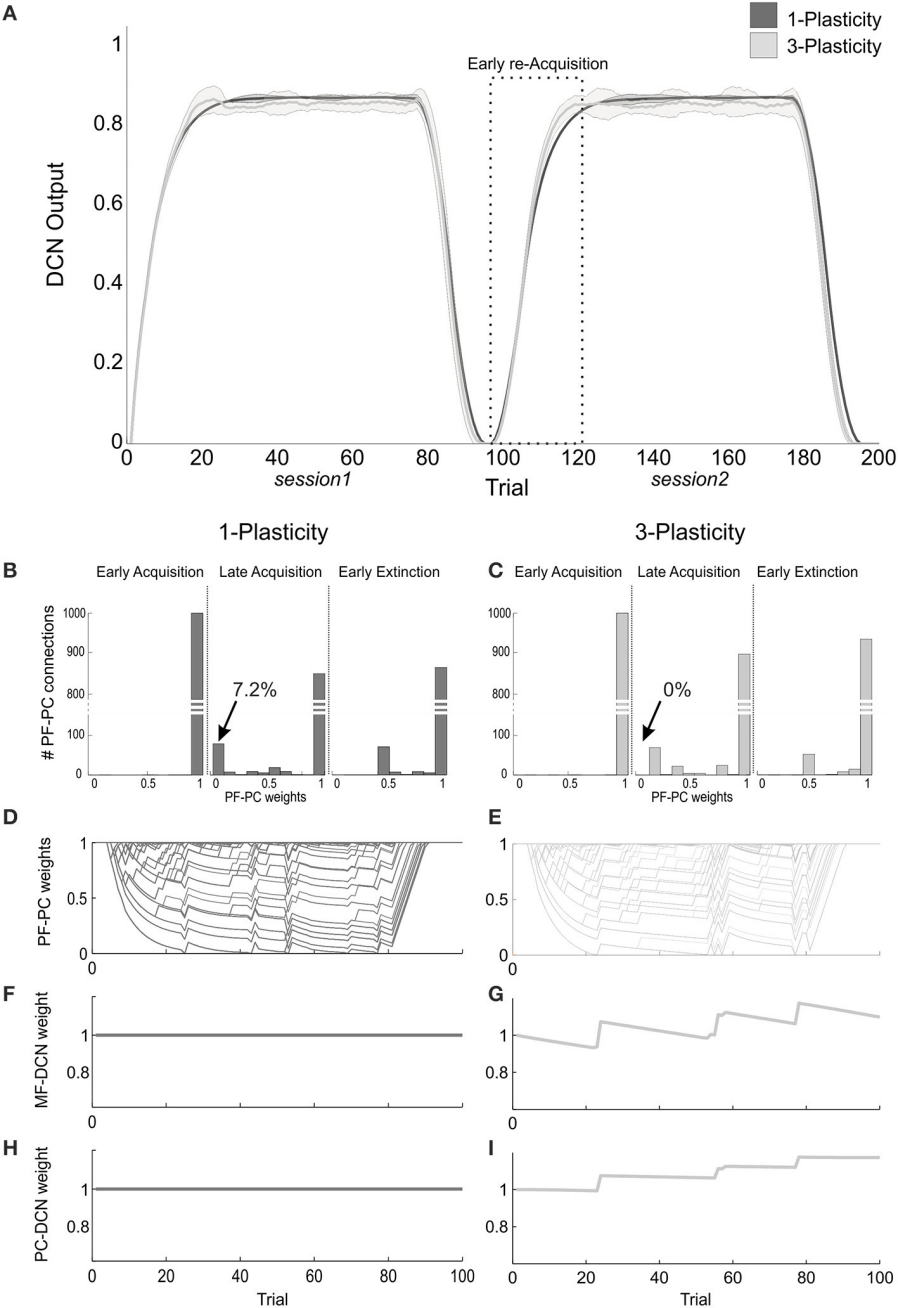
**Plasticity roles in real-robot Pavlovian task. (A)** The two-session Pavlovian task, with ISI_2._ The curves are the maximum DCN output within each trial, averaged on the 20 tests, and the areas are the standard deviations. Dark gray: 1-plasticity model; light gray: 3-plasticity model. **(B,C)** For each model, histograms of the PF-PC weights at the end of three trials (1st, 80th, and 85th), in one of the 20 tests. The arrows indicate the percentages of saturated LTD at PF-PC synapses (weights = 0) at the 80th trial. For the 1-plasticity model, a considerable number of PF-PC connections is saturated to zero, on the contrary for the 3-plasticity model none of the connections is saturated. **(D,E)** For each model, PF-PC weights at the end of each trial of the test. Here each of the 1000 PF-PC synapses corresponds to one line (there are a lot of overlap, i.e., PF-bundles, as explained in the Results). **(F,G)** For each model, MF-DCN weight at the end of all trials of the test. The value is fixed for the 1-plasticity model. **(H,I)** For each model, PC-DCN weight at the end of all trials of the test. The value is fixed for the 1-plasticity model.

When comparing the two models in terms of ΔDCN_s1_, ΔDCN_s2_, and CR latency for each ISI, a significant difference came out only for the ΔDCN_s2_ in all the three ISIs (Figure 4): in ISI_1_, *t* = −5.8; *p* = 9.3289e-07; in ISI_2_, *t* = −5.8; *p* = 1.2288e-06; in ISI_3_, *t* = −4.6; *p* = 4.0573e-05. Hence, the learning in the re-testing phase was significantly faster when the neurorobot was controlled by the 3-plasticity model than by the 1-plasticity model (Figure 5A).

The modulation of each plastic connection embedded into the cerebellar models represents the intrinsic mechanisms underlying these observed behaviors. For each trial, the PF-PC synapses that were activated without any correlated CF-signal reaching the PC underwent LTP, whereas the PF-PC synapses activated at the time-states of the movement when a signal arrived to the PC from CF developed LTD. As the trial-by-trial variability, LTD and LTP did not develop alike in fixed bundles of PF-PC connections; the most of PFs decoded system time-states outside the US timeframe; indeed, the US lasted 20% of the whole trial duration. Therefore, these synapses maintained maximum values (saturated LTP); the PFs always decoding system time-state during US occurrence underwent an equal strong LTD; the few PFs at the US time-window borders underwent different balance LTD/LTP, thus they spread across the weight ranges (0–1) (Figures 5B–E).

The main phenomenon driving acquisition was the development of LTD at the PF-PC synapses. In the 3-plasticity model, in the meanwhile, with a slower rate, plasticity at the MF-DCN synapse and at the PC–DCN synapse occurred (Medina et al., 2000) (Figures 5F–I): the nuclear sites evolved taking charge of a part of the activity generating output responses, which initially was entirely due to the cortical plasticity effects. In other words, a partial transfer of output activity occurred from cortical to nuclear plasticity sites. In session1, this slow transfer did not change any overall learning performances. The network was able to rapidly extinguish the stimuli association by fast PF-PC LTP, but without canceling the slower nuclear plastic changes which had occurred. At the first trials of the session2, the cerebellar synapses of the 3-plasticity model were in an effectively different state compared to the synapses of the 1-plasticity model: the distributed plasticity dynamics, able to store information, was responsible for the higher learning rate in session 2.

### VOR task

**Figure 6:**
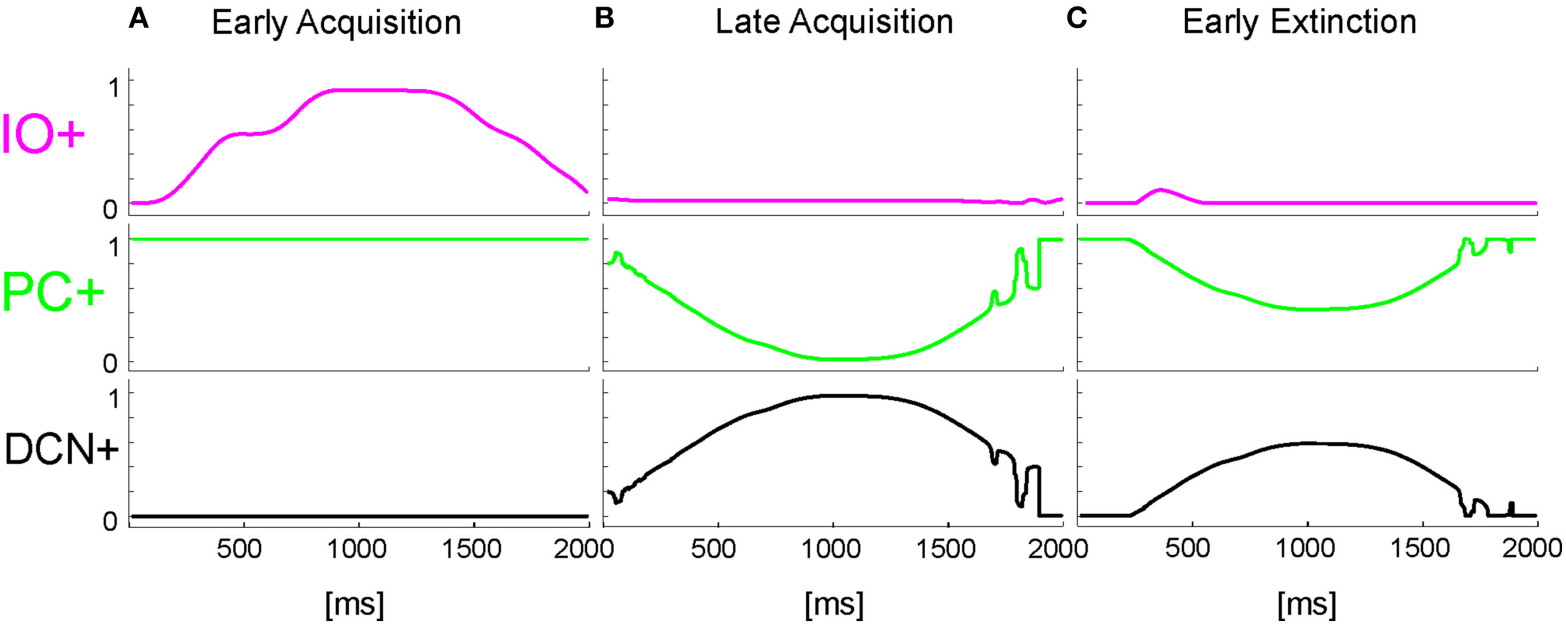
**Real-robot VOR task**. Three exemplificative trials of the session1 (40 trials of acquisition + 20 trial of extinction) of VOR task carried out by the neurorobot are described by displaying the IO, PC, and DCN activity. **(A)** 1st trial, **(B)** 40th trial, **(C)** 45th trial.

The onset of the vestibular stimulus, i.e., the onset of MF activity, initiated the generation of the state coding within the GR layer, and also provided the excitatory drive to DCN cells. The decoding of the gaze error reached continuously the Purkinje cells through the IOs. The Purkinje cells in turn inhibited the DCNs. At the beginning of the acquisition phase, the Purkinje cells were spontaneously active, supplying tonic inhibition to the DCNs (Figure 6A). After acquisition, PC+ activity was decreased. Summing up all the presynaptic (constant or plastic) inputs to DCN+, DCN+ neurons began to fire so as to continuously counterbalance the head movement, minimizing the gaze error (Figure 6B). Then during extinction trials, PC activity was progressively re-increased; and DCN+ decreased the output motor commands actuating eye motion (Figure 6C).

In each condition (three different HRs), the maximum HR came out not perfectly constant, both across trials of the same test and across the 15 tests (HR_1_ 1-plast: 24 ± 0.8; HR_1_ 3-plast: 24.8 ± 2; HR_2_ 1-plast: 29.2 ± 0.8; HR_2_ 3-plast: 30 ± 1; HR_3_ 1-plast: 36.9 ± 0.7; HR_3_ 3-plast: 36.2 ± 1.1). This variability was due to the inertial components of the robot. In each condition, no statistical difference came out between the resulting HR which 1-plasticity and 3-plasticity models were subjected to. Thus, as for the EBCC task, the necessary prerequisite about the same experimental conditions between the two cerebellar models was verified, so allowing us to ascribe possible differences between their behavioral outcomes to the different neural mechanism functioning.

The One-Way ANOVA tests analyzed whether the experimental condition (3 levels of HR) affected the outcome behavior for each cerebellar model. Both for the 1-plasticity model and for the 3-plasticity model, HR value affected the two learning rates (ΔDCN_s1_ 1-plast: *F* = 63167, *p* = 0; ΔDCN_s1_ 3-plast: *F* = 24433, *p* = 0; ΔDCN_s2_ 1-plast: *F* = 261, *p* = 0; ΔDCN_s2_ 3-plast: *F* = 6150, *p* = 0). Higher was the head perturbation, faster was the DCN activity modulation.

**Figure 7:**
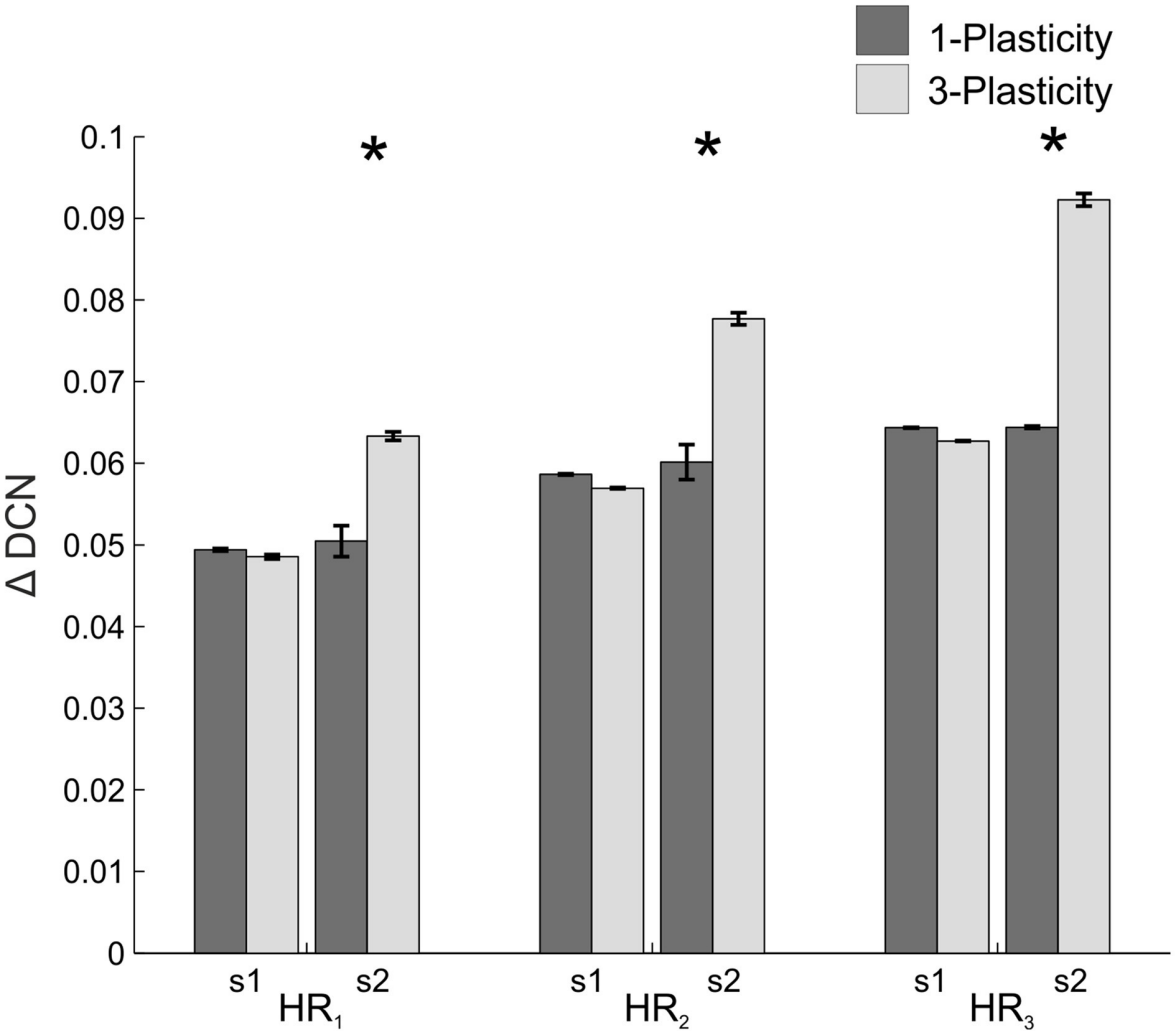
**Real-robot two-session VOR task: models comparison**. The histogram reports the performances (ΔDCN) of the 1-plasticity and 3-plasticity models in the three VOR conditions (HR_1_, HR_2_, and HR_3_) in both sessions (s1 and s2). Bars indicate the mean across the 15 tests and the relative standard deviation. ^*^Means significant statistical difference between the two cerebellar models (*p* < 0.01).

When comparing the two models in terms of ΔDCN_s1_ and ΔDCN_s2_ for each HR, a significant difference came out only for the ΔDCN_s2_ in all the three HRs (Figure 7): in HR_1_, *t* = −24.4; *p* = 0; in HR_2_, *t* = −29; *p* = 0; in HR_3_, *t* = −132; *p* = 0. Hence, the learning in the re-testing phase was significantly faster when the neurorobot was controlled by the 3-plasticity model than by the 1-plasticity model.

**Figure 8:**
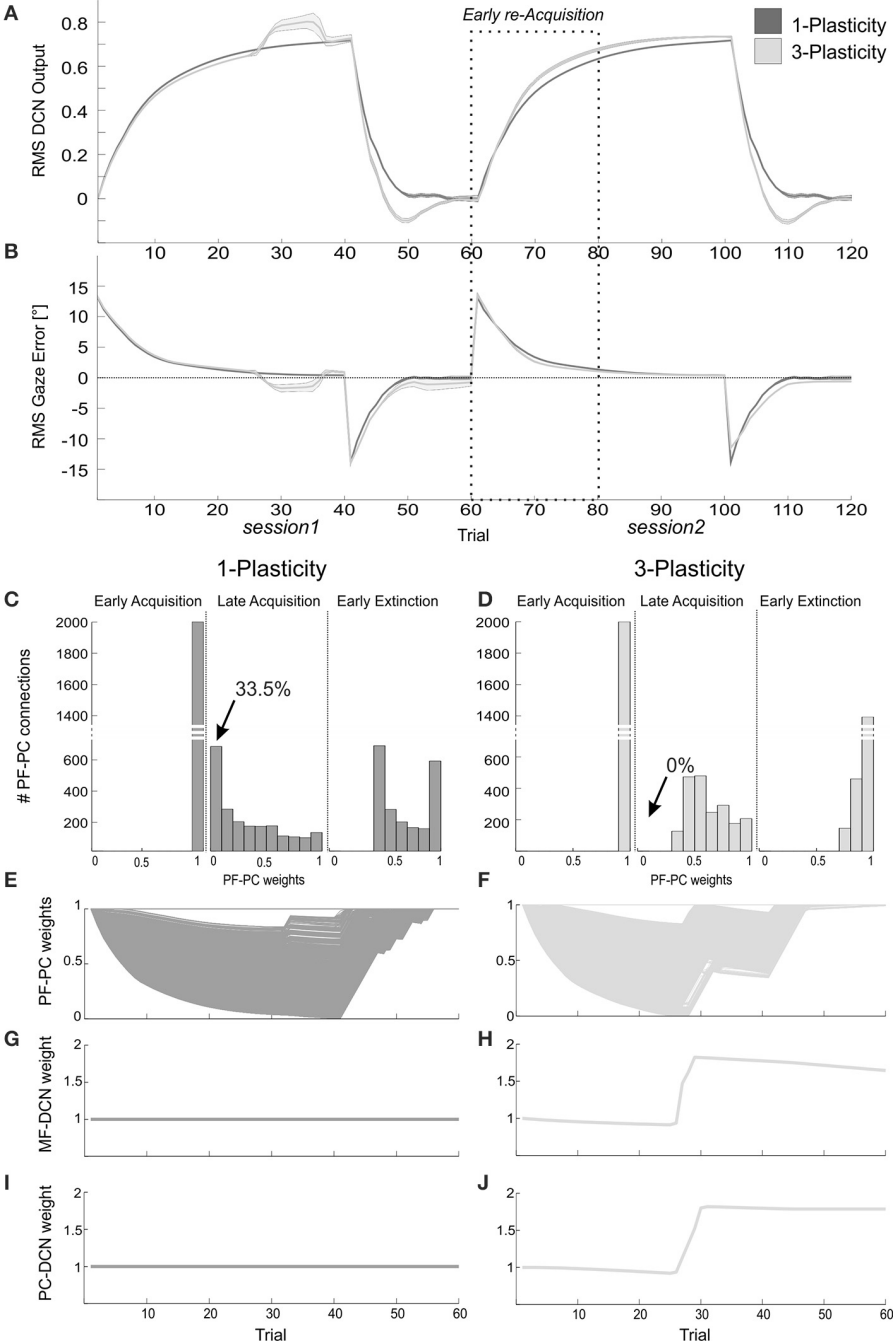
**Plasticity roles in real-robot VOR task. (A)** The two-session VOR task, with HR_1._The curves are the RMS DCN output within each trial, averaged on the 15 tests, and the areas are the standard deviations. Dark gray: 1-plasticity model; light gray: 3-plasticity model. **(B)** The corresponding RMS gaze error within each trial, taking into account the error sign. **(C,D)** For each model, histograms of the PF-PC+ weights at the end of three trials (1st, 40th, and 45th). in one of the 15 tests. The arrows indicate the percentages of saturated LTD at PF-PC synapses (weights = 0) at the 40th trial. For the 1-plasticity model, a considerable number of PF-PC connections is saturated to zero, on the contrary for the 3-plasticity model none of the connections is saturated. **(E,F)** For each model, PF-PC+ weights at the end of all trials of the test. Here each of the 2000 PF-PC synapses corresponds to one line (there are a lot of overlap, i.e., PF-bundles, as explained in the Results). **(G,H)** For each model, MF-DCN+ weight at the end of each trial of the test. The values are fixed for the 1-plasticity model. **(I,J)** For each model, PC+-DCN+ weight at the end of all trials of the test. The values are fixed for the 1-plasticity model. For picture clarity, we report only the weights at the DCN+, which is the only output cell involved during the acquisition phases, since the positive sign of the gaze error (in extinction for few trials, during the after-effects, DCN- is slightly involved).

The DCN output of the 3-plasticity model showed an evolution across sessions, while the DCN output of the 1-plasticity model repeated exactly the same adaptation process regardless any previous achieved acquisition (Figure 8A).

Since the functioning of the cerebellum as predictive controller acting based on previous trials, during the extinction phases the after-effects occurred for few repetitions: even if the head rotation was canceled, the network output still produced eye compensation; this overcompensation led to a gaze error with opposite sign (Figure 8B). Rapidly, the network learned to bring back the error to zero level.

The modulation of each plastic connection embedded into the cerebellar models represents the intrinsic mechanisms underlying these observed behaviors. For each trial, the PF-PC synapses that were activated without any correlated CF-signal reaching the PC underwent LTP, whereas the PF-PC synapses activated at the time-states of the movement when a signal arrived to the PC from CF developed LTD. Sequentially all the 2000 PFs decoded system time-state during head motion, therefore corresponding to a not-null but not constant gaze error; they underwent a proportional LTD, hence spreading across the weight range (0–1) (Figures 8C–F). The PF-PC weight histograms (Figures 8C,D) clearly showed that in late acquisition the same behavioral outcomes, i.e., steady eye motion fully compensating head motion, was achieved by different weight distributions between the two models. Most of these weights in the 1-plasticity controller was saturated at 0 level; whereas, in the 3-plasticity one, they were more distributed around half value of their range. The main phenomenon driving acquisition was the development of LTD at the synapses PF-PC; however, in the 3-plasticity model, in the meanwhile, with a slower rate, plasticity at the MF-DCN synapse and at the PC–DCN synapse occurred (Medina et al., 2000) (Figures 8G–J). Thus, in the same way as in the EBCC task, a partial transfer of activity responsible for motor response generation occurred from cortical to nuclear plasticity sites. These changes of weights at DCN sites led to a partial release of the cortical synapses. The network was then able to decrease the eye motion by fast PF-PC LTP, but without canceling the slower nuclear plastic changes had occurred. Again the distributed plasticity dynamics, able to store information, was responsible for the higher learning rate in session2.

**Figure 9:**
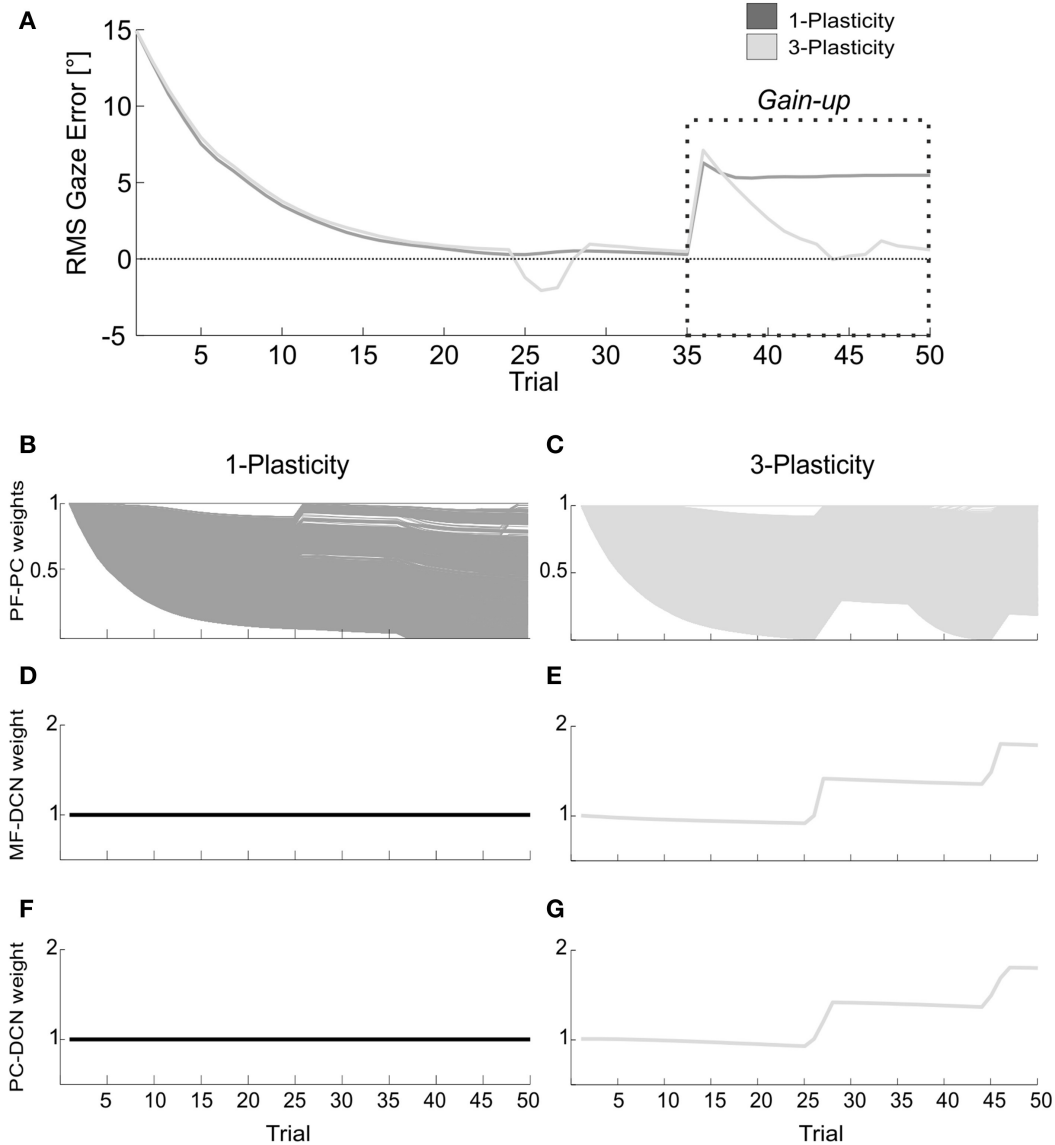
**Real-robot gain-up VOR task. (A)** The gain-up VOR test, with HR_1_ from 1st to 35th trials and then 150% of HR_1_ for the following 15 trials. The curves report the RMS gaze error within each trial, taking into account the error sign. Dark gray: 1-plasticity model; light gray: 3-plasticity model. **(B,C)** For each model, PF-PC+ weights at the end of all trials of the test. Here each of the 2000 PF-PC synapses corresponds to one line. **(D,E)** For each model, MF-DCN+ weight at the end of all trials of the test. The values are fixed for the 1-plasticity model. **(F,G)** For each model, PC+-DCN+ weight at the end of all trials of the test. The values are fixed for the 1-plasticity model. For picture clarity, we report only the weights at the DCN+, which is the only output cell involved in this acquisition test.

The memory transfer effect pointedly arose in the gain-up VOR test (Figure 9). Indeed, the passage from cortical to nuclear sites made the PF-PC synapses ready for further plasticity. In this way, they were able to react to other additive perturbations, suddenly presented to the system. In late acquisition, the performances of the two models were comparable, but the PF-PC synapses of the 1-plasticity controller were close to saturation. When the gain-up stimulus was provided, the 1-plasticity model exploited the residual cortical plasticity till complete saturation; it did not lead to an accurate eye compensatory movement. Whilst, the 3-plasticity model exploited the more persistent nuclear changes and the more availability of plasticity at cortical level; this efficient plasticity interaction led to an accurate recalibration of the eye motion.

## Discussion

In this study, the developed cerebellar scheme, equipped with cortical and nuclear plasticity mechanisms (Garrido et al., 2013), has been transformed into a real-time controller of cerebellar-mediated tasks in real-world, EBCC-like and VOR protocols. In this way, it allowed us to challenge the realistic learning properties of the model in uncertainty conditions, in which inputs repeatability was not guaranteed along trials within each test and along multiple tests. By varying stimuli patterns, control robustness has been investigated. Through the designed protocols, we have shed light on acquisition, extinction and consolidation mechanisms, credited to the different active plasticity sites, and we have tested the generalization capability of the modeled computational mechanisms in learning both associative discrete responses and continuously tuned motor responses. The cerebellar controller equipped with cortical and nuclear plasticity mechanisms proved superior to single-site plasticity in developing consolidation process and memory transfer and in implementing adaptable gain control facing varying operative ranges.

The model we have customized and embedded here in the neurorobot was previously developed as a general computational scheme and tested in perfectly repeatable *in-silico* simulations. Moreover, the tracking task carried out by a simulated robotic arm did not represent a cerebellum-based learning paradigm.

The model was built on the assumption that there are three main cerebellar learning sites, one in the cerebellar cortex (PF-PC) and two in the DCN (MF-DCN and PC-DCN), all generating LTP and LTD with site-specific dynamics.

Recently, neurophysiological studies (Masuda and Amari, 2008) proposed that MF-DCN synapses or PC-DCN synapses are plastic on a slow time scale and store permanent memory, whose content is passed from the cerebellar cortex storing transient memory.

Clear evidences from mouse mutants (De Zeeuw and Yeo, 2005) showed similarities in EBCC and VOR behaviors: postsynaptic parallel fibers LTD is the main responsible for adaptation; whereas postsynaptic parallel fibers LTP is responsible for decreasing VOR gain and for driving EBCC extinction. Since spontaneous recovery of the original response and faster relearning (“savings” effect) observed in human behaviors, they suggested that other forms of plasticity may contribute when longer time periods are available. One of the interesting candidates for this mechanism is the firing rate modulation in the deep nuclei. Their long-lasting changes in intrinsic excitability, which are relatively difficult to reverse, make this form of plasticity well suited for chronic motor learning and persistent memory.

The behavioral fall-outs of this model emerged in our tests. To our knowledge, it is the first time an embodied distributed realistic cerebellar model, tested in cerebellum-mediated paradigms, came across able to robustly reproduce human-like effective learning properties in acquisition, extinction and re-acquisition, dealing with different external and noisy stimuli in real-world.

In the Pavlovian task, the neurorobot expressed response levels comparable to those found in human EBCC with similar ISIs, where a stable behavior was achieved in about 30 trials (Bracha et al., 2000; Hoffland et al., 2012; Monaco et al., 2014). Concerning the VOR task, neurophysiological studies showed how in a visual-vestibular training the cerebellum functioning led to an image slip minimization around 0.2° (Kimpo et al., 2005).

The 3-site model revealed itself in the motor memory transfer between cerebellar sites; in this way, the cerebellar model was equipped with the intrinsic capability to optimize the learning on multiple time-scales and to effectively adapt to dynamic ranges of stimuli.

These outcomes are consistent with the hypothesis about the coexistence of two processes proceeding at different rates in the cerebellum-mediated learning and located in different cerebellar sites, cerebellar cortex and deep cerebellar structures (Medina et al., 2001; Smith et al., 2006). The fast process was made dominant by large errors, while the slow process by small errors. The existence of a fast rapidly reversible learning process emerged during the early acquisition and extinction phases. The existence of a slower process emerged in late acquisition. When re-tested, the neurorobot seemed to partially exploit previous learned skills; indeed, the extinction phase of the first session did not reset all the DCN plastic changes achieved in the acquisition training.

In summary, we have linked low-level mechanisms of the cerebellar circuit with high-level functions, by integrating a detailed adaptive cerebellar controller into a neurorobot sensing and operating in real-world. The embedded plasticity dynamics were reflected in behavioral tasks: the fundamental aspects of cerebellar function—prediction, learning, timing and memory—were generated (D'Angelo et al., 2013).

As a further advance, the platform could be updated with new neurophysiological properties, such as the IO-DCN excitatory connection, working on a much faster timescale than the ones embedded in the present neural model (Luque et al., 2014). Furthermore, the distributed plasticity model could be translated into a more realistic spike-timing computational scheme (Casellato et al., 2014).

It is envisaged that improving the realism of the model will allow us to make predictions about the nature of implicit computations occurring in the cerebellar circuits; it could represent a precious tool to simulate neural dysfunctions and thus to predict behaviors, or viceversa to reproduce observed misbehaviors and thus to predict underlying dysfunctions; the platform can be easily manipulated to generate and test any conditions to associate neural features with explicit functions.

### Conflict of interest statement

The authors declare that the research was conducted in the absence of any commercial or financial relationships that could be construed as a potential conflict of interest.

## Abbreviations

CF: Climbing Fiber
CR: Conditioned Response
CS: Conditioned Stimulus
DCN: Deep Cerebellar Nucleus
EBCC: EyeBlink Classical Conditioning
GR: Granular cell
HR: Head Rotation
IO: Inferior Olive cell
ISI: Inter-Stimuli Interval
LTD: Long-Term Depression
LTP: Long-Term Potentiation
MF: Mossy Fiber
PC: Purkinje Cell
PF: Parallel Fiber
RMS: Root Mean Square
US: Unconditioned Stimulus
VOR: Vestibulo-Ocular Reflex.
